# Effects of eating dinner alone on overweight in Japanese adolescents: a cross-sectional survey

**DOI:** 10.1186/s12887-018-1041-y

**Published:** 2018-02-07

**Authors:** Takako Shirasawa, Hirotaka Ochiai, Takahiko Yoshimoto, Masaaki Matoba, Yuma Sunaga, Hiromi Hoshino, Akatsuki Kokaze

**Affiliations:** Department of Public Health, Showa University School of Medicine, 1-5-8 Hatanodai, Shinagawa-ku, Tokyo, 142-8555 Japan

**Keywords:** Eating dinner alone, Overweight, Adolescents, Japan

## Abstract

**Background:**

The decrease in the frequency of family meals among Asian youth is often lamented. In Japan, adolescents who eat breakfast alone might have an unhealthy diet, which increases the risk of overweight or obese. However, there are few studies on the relationship between eating dinner alone and overweight in Japanese adolescents. Here, we investigated if eating dinner alone is associated with being overweight in Japanese adolescents of each sex.

**Methods:**

The participants consisted of 890 seventh graders (12–13 years of age) from the junior high schools of Ina, Japan who were recruited from 2011 to 2012. Information about eating dinner alone was obtained using a self-reported questionnaire, which was given to each participant. The participants were classified into the following three groups: does not eat alone, eats alone 1–2 times/week, or eats alone ≥3 times/week. A logistic regression model was used to examine the relationship between eating dinner alone and being overweight. The height and weight of each participant were measured. Childhood overweight status was defined using the body mass index cutoff points proposed by the International Obesity Task Force.

**Results:**

When compared with girls who did not eat dinner alone, a significantly increased odds ratio (OR) was observed among girls who ate dinner alone ≥1 time/week (adjusted OR = 2.78; 95% confidence interval = 1.21–6.38). In contrast, there was no statistically significant difference between eating dinner alone and being overweight among boys.

**Conclusion:**

The present study found that eating dinner alone is associated with being overweight among adolescent girls in this community in Japan. Therefore, reducing the frequency of eating dinner alone might contribute to decreasing the risk for becoming overweight or obese among adolescent girls.

## Background

Childhood obesity is one of the most serious public health challenges of the twenty-first century [1], and the increase in obesity is possibly associated with inappropriate dietary habits [2]. Previously published studies report that eating family meals together is a potential factor that may protect against unhealthy eating and obesity during childhood and adolescence [3–9]. Increasing the frequency of eating family meals together may decrease the risk of overweight or obese in children and adolescents [8]. A meta-analysis suggested that children and adolescents in families that share ≥3 family meals/week are 12% less likely to be overweight in comparison with those who share < 3 family meals/week [4]. A previous study reported that eating meals as a family every day is associated with a lower rate of obesity, as well as good lifestyle habits such as eating balanced meals [5]. Thus, the family meal is important for preventing overweight and obesity in children and adolescents.

However, a recent study showed a decrease in the frequency of family meals among Asian youth between 1999 and 2010 [10]. In Japan, Adachi reported that 12.6% of children eat their breakfast with all members of their family, and this is a 10% decrease compared with a similar study that was conducted approximately 20 years ago (in 1981) [11, 12]. In addition, Adachi reported that 26.4% of children eat breakfast alone, 24.5% eat with other children but no adults, and these two groups combined showed a 12% increase compared with results reported 20 years ago [11, 12]. Moreover, the increase in the proportion of children eating alone is often lamented in the popular media in Japan [13].

Previous studies reported that adolescents who eat breakfast alone tend to have a low intake of vegetables and fish in their daily diet, thereby suggesting unhealthy dietary habits [14, 15]. Therefore, adolescents who eat breakfast alone might have an unhealthy diet, which increases the risk of overweight or obese. However, to the best of our knowledge, there are few studies on the relationship between eating dinner alone and being overweight in Japanese adolescents [16, 17].

Accordingly, here we investigated if eating dinner alone is associated with being overweight in Japanese adolescents of each sex. Our hypothesis was that a high frequency of eating dinner alone would be associated with being overweight in adolescent boys and girls.

## Methods

### Participants

In addition to the annual national health checkups that are performed in accordance with the School Health Law of Japan, the town of Ina in the Saitama Prefecture of Japan runs a health promotion program as a part of its community health services. As a part of this program, a questionnaire survey and physical examinations are administered to seventh graders. The present study was conducted as a part of this program.

The study participants in the present study consisted of seventh graders (12–13 years of age) who were recruited from the junior high schools of the town of Ina, Japan in 2011–2012 (*n* = 890). Written informed consent was obtained from each child’s parent or guardian. The Medical Ethics Committee of Showa University School of Medicine approved the study protocol (No.127).

### Questionnaire survey

The following information was obtained using a self-reported questionnaire that was given to each child: sex, age, skipping breakfast (yes vs no), dinner time (regular vs irregular), eats dinner alone (yes vs no), snacks after dinner (yes vs no), eating speed (fast, medium, or slow), eats until full (yes vs no), and sports teams’ participation (yes vs no). Each participant’s parent or guardian was asked to complete a self-administered questionnaire regarding the number of siblings of each participant (only child vs ≥ 1 siblings) and paternal and maternal employment (yes vs no).

Eating dinner alone was assessed using the following question on the self-administered questionnaire that was given to each child: “Do you eat dinner alone?” If the child answered “yes” to this question, the frequency of eating dinner alone was evaluated using the following question: “How many times do you eat dinner alone during 1 week?” Based on these answers, we categorized the participants into three groups: does not eat alone, eats alone 1–2 times/week, or eats alone ≥3 times/week.

### Anthropometric measurements

Measurements of the height and weight of each child were performed annually during 2011–2012. The same examination protocol was used annually to ensure uniform quality and precise assessments. For all measurements, all children were asked to remove their shoes and socks, after which their height and weight were measured in increments of 0.1 cm and 0.1 kg, respectively, while they were wearing light clothing. Body mass index (BMI) was calculated as the participant’s body weight (kg) divided by the square of their height (m^2^). Childhood overweight status was defined using the BMI cutoff points proposed by the International Obesity Task Force (i.e., the age- and sex-specific cutoff points that are linked to a BMI value of 25 at 18 years of age) [18]. The BMI cutoff points are based on average data collected from six countries (including Asian countries), and these cutoff points were shown to be applicable to Japanese children [19]. In the present study, the BMI cutoff points for childhood overweight status were 21.22 (age 12) and 21.91 (age 13) for boys and 21.68 (age 12) and 22.58 (age 13) for girls. For the purposes of the present study, obese was included as overweight. Children who were not overweight were regarded as non-overweight. Therefore, each participant in this study was categorized into one of two weight status categories (non-overweight vs overweight).

### Statistical analysis

The unpaired t-test or chi-squared test was used to compare the characteristics between boys and girls. In the analysis stratified by sex, a logistic regression model was used to examine the relationship between eating dinner alone and being overweight. Because the proportions of overweight boys and girls who either ate alone ≥3 times/week and 1–2 times/week were small, we combined these categories into a single category (eating dinner alone ≥1 time/week) and used propensity score adjustment in the model. The propensity score is the conditional probability of being exposed given the observed covariates [20, 21], and propensity score adjustment preserves statistical power by reducing covariates into a single variable [22]. In this study, the observed covariates were age, being an only child, paternal employment, maternal employment, sports teams’ participation, skipping breakfast, dinner time, snacking after dinner, eating speed, and eating until full, which have been reported to related to being overweight [19, 23]. In the model, crude odds ratios (ORs) and 95% confidence intervals (CIs) for being overweight were first estimated and subsequently adjusted for the propensity score. In this study, *P* <  0.05 was considered statistically significant. All data were analyzed using SPSS 20.0 J (IBM, Chicago, Illinois, USA) and JMP Pro 13 (SAS Institute Inc., Cary, North Carolina, USA).

## Results

Among the 890 participants, 5 participants refused to participate in the program (participation rate = 99.4%) and 5 participants were excluded because of incomplete data. Therefore, a total of 880 participants (470 boys and 410 girls) were analyzed in the present study.

The characteristics of the study participants (boys and girls) are shown in Table 1. The proportion of overweight boys was significantly higher than among girls. Although the proportion of participants who ate dinner alone was 14.7% among boys (6.0% and 8.7% reported eating alone ≥3 times/week and 1–2 times/week, respectively) and 10.8% among girls (4.9% and 5.9% reported eating alone ≥3 times/week and 1–2 times/week, respectively), the difference in the proportions between boys and girls did not reach a significant level. Moreover, statistically significant differences between boys and girls were observed in terms of maternal employment, sports teams’ participation, dinner time, eating speed, and eating until full.

**Table 1:** Characteristics of the study participants

Variable	Boys (*n* = 470)	Girls (*n* = 410)	*P* ^a^
Age (years) (mean ± SD)	12.1 ± 0.3	12.1 ± 0.3	0.424
Height (cm) (mean ± SD)	151.9 ± 8.1	151.8 ± 5.9	0.764
Weight (kg) (mean ± SD)	42.9 ± 9.5	42.9 ± 8.1	0.887
BMI (kg/m^2^) (mean ± SD)	18.4 ± 2.9	18.6 ± 2.8	0.468
Actual weight status (%)			
Overweight	15.7	10.0	0.012
Non-overweight	84.3	90.0	
Only child (%)	14.7	12.5	0.335
Paternal employment (%)	93.6	95.1	0.348
Maternal employment (%)	65.9	72.1	0.049
Sports teams’ participation (%)			
Yes	79.1	48.7	< 0.001
No	20.9	51.3	
Skipping breakfast (%)			
Yes	0.6	2.0	0.080
No	99.4	98.0	
Dinner time (%)			
Regular	89.3	93.6	0.023
Non-regular	10.7	6.4	
Eats dinner alone (%)			
None	85.3	89.3	0.192
1–2 /week	8.7	5.9	
≥3 /week	6.0	4.9	
Snacks after dinner (%)			
Yes	49.4	52.6	0.342
No	50.6	47.4	
Eating speed (%)			
Fast	23.2	13.4	< 0.001
Medium	59.9	62.1	
Slow	16.8	24.4	
Eats until full (%)			
Yes	70.7	64.4	0.047
No	29.3	35.6	

*SD* standard deviation, *BMI* body mass index

^a^Determined using the unpaired t-test or chi-squared test

The characteristics of the overweight and the non-overweight participants by sex are shown in Table 2. Statistically significant differences between the overweight and the non-overweight participants were observed in terms of the eating speed for each sex. The proportion of participants who ate fast was higher in the overweight group than in the non-overweight group regardless of sex. Among boys, the proportion of participants who were only children was higher in the overweight group than in the non-overweight group. A statistically significant difference was not observed in terms of eating dinner alone between overweight and non-overweight boys. In contrast, a statistically significant difference between the overweight group and the non-overweight group was found among the female participants who ate dinner alone; the proportion of participants who ate dinner alone in the overweight group was higher than in the non-overweight group.

**Table 2:** Characteristics of the non-overweight and the overweight participants by sex

	Boys (*n* = 470)			Girls (*n* = 410)		
Variable	Overweight (*n* = 74)	Non-overweight (*n* = 396)	*P* ^a^	Overweight (*n* = 41)	Non-overweight (*n* = 369)	*P* ^a^
Age (years) (mean ± SD)	12.1 ± 0.3	12.1 ± 0.3	0.383	12.1 ± 0.3	12.1 ± 0.3	0.688
Height (cm) (mean ± SD)	155.9 ± 7.9	151.2 ± 7.9	< 0.001	154.6 ± 4.7	151.5 ± 6.0	< 0.001
Weight (kg) (mean ± SD)	57.8 ± 8.7	40.1 ± 6.5	< 0.001	58.7 ± 7.6	41.2 ± 6.0	< 0.001
BMI (kg/m^2^) (mean ± SD)	23.7 ± 2.4	17.4 ± 1.6	< 0.001	54.5 ± 2.8	17.9 ± 1.9	< 0.001
Only child (%)	25.7	12.7	0.004	9.8	12.8	0.579
Paternal employment (%)	88.9	94.5	0.077	92.7	95.3	0.458
Maternal employment (%)	58.3	67.3	0.142	70.7	72.3	0.837
Sports teams’ participation (%)						
Yes	85.1	78.0	0.167	42.5	49.3	0.412
No	14.9	22.0		57.5	50.7	
Skipping breakfast (%)						
Yes	1.4	0.5	0.401	4.9	1.6	0.153
No	98.6	99.5		95.1	98.4	
Dinner time (%)						
Regular	86.5	89.8	0.390	95.1	93.5	0.682
Non-regular	13.5	10.2		4.9	6.5	
Eats dinner alone (%)						
None	83.8	85.6	0.776	78.0	90.5	0.033
1–2 /week	10.8	8.3		9.8	5.4	
≥ 3 /week	5.4	6.1		12.2	4.1	
Snacks after dinner (%)						
Yes	45.9	50.0	0.522	56.1	52.2	0.634
No	54.1	50.0		43.9	47.8	
Eating speed (%)						
Fast	47.3	18.7	< 0.001	24.4	12.2	0.032
Medium	50.0	61.8		63.4	62.0	
Slow	2.7	19.5		12.2	25.8	
Eating until full (%)						
Yes	75.7	69.7	0.302	65.0	64.3	0.931
No	24.3	30.3		35.0	35.7	

^a^Determined using the chi-squared test

Next, the crude and adjusted ORs of eating dinner alone for the overweight participants were calculated for each sex, as shown in Table 3. When compared with girls who did not eat dinner alone, a significantly increased OR was observed among the girls who ate dinner alone ≥1 time/week (adjusted OR = 2.78; 95% CI = 1.21–6.38). In contrast, there was no statistically significant difference between eating dinner alone and being overweight among boys.

**Table 3:** Association between eating dinner alone and being overweight by sex

	Total	Overweight	Crude		Adjusted^a^	
Eating dinner alone	N	n (%)	OR	95% CI	OR	95% CI
Boys (*n* = 470)						
None	401	62 (15.5)	1.00		1.00	
≥ 1 /week	69	12 (17.4)	1.15	0.58–2.27	0.95	0.46–1.95
Girls (*n* = 410)						
None	366	32 (8.7)	1.00		1.00	
≥ 1/week	44	9 (20.5)	2.68	1.19–6.08	2.78	1.21–6.38

*OR* odds ratio, *95% CI* 95% confidence interval

^a^Adjusted for the propensity score

## Discussion

The present study shows that the proportions of children who eat dinner alone ≥3 times/week were 6.0% among boys and 4.9% among girls. According to a survey of schoolchildren in Japan (performed in 2010), 6.0% of junior high school children eat dinner alone ≥3 times/week, which is similar to the results of our study [24]. Eating alone is believed to lead to unhealthy dietary habits because such adolescents are not supervised by their guardians and consequently often eat only the foods they like [15]. Therefore, it is important to consider eating alone as a serious health problem among children and adolescents.

In this study, eating dinner alone was significantly associated with being overweight among girls. One explanation for this result is that eating dinner alone might lead to unhealthy dietary habits, thereby resulting in being overweight. Previous studies have reported that family meal times may act as a protective factor against many nutritional health-related problems encountered during childhood and adolescence, including issues related to being overweight, unhealthy eating, and disordered eating [4, 8]. In addition, Feldman et al. reported that families who choose to eat together might be more likely to try to prepare well-balanced, nutritious meals in comparison with adolescents who eat on their own and may rely on prepackaged convenience foods for meals, which often lack fruits and vegetables [25]. Therefore, our study findings suggest that eating dinner alone leads to adolescents being overweight.

Furthermore, in the present study, we report the sex differences among adolescents in terms of the association between eating dinner alone and being overweight: compared with girls who did not eat dinner alone, those who ate dinner alone ≥1 time/week were at a greater risk of being overweight, but there was no statistically significant relationship between eating dinner alone and being overweight among boys. These results remained even after propensity score adjustment. A previous study reported that, among girls, the intake of food items such as fish and vegetables significantly differed between those who always ate dinner with their family and those who rarely ate dinner with their family, but among boys there was no significant difference in food intake depending on family eating habits [26]. Neumark-Sztainer et al. suggested that regular family meals during adolescence plays a protective role against extreme weight control behaviors and disordered eating behaviors in adolescent girls, but not boys [27]. Moreover, boys’ mothers tend to prepare meals for their sons, and girls’ mothers usually encourage their daughters to cook for themselves [26]. Therefore, when adolescent boys and girls eat dinner alone, boys might eat meals prepared by their mothers, whereas girls might freely cook their favorite menu items, thereby resulting in an unhealthy diet and eating behaviors. In this study, the proportion of participants who eat until full, which has been reported to be associated with being overweight [28], was significantly higher among girls who eat dinner alone (50% and 55.0% of girls eat until full among girls who eat dinner alone 1–2 times/week and ≥3 times/week, respectively) than in girls who do not eat dinner alone (33.6% of girls who do not eat dinner alone also eat until they are full) (*P* = 0.048; data not shown). Therefore, it could be important to reduce the frequency of eating dinner alone in order to promote healthy eating behaviors and prevent overweight status among adolescents, especially girls.

The present study also has some limitations. First, the questionnaire used in our study was not validated. Therefore, to verify our study results, a validated questionnaire should be used in future research. Second, this study did not consider factors in the home environment such as watching television during meals [8, 25]. For example, watching television during family meals is associated with poorer dietary quality among adolescents [25]. Therefore, these factors could have affected our study findings. Third, the participants in this study were seventh graders (12–13 years of age) from a single town in Japan, which might limit generalizability to other countries. Finally, the present study could not determine a causal relationship between eating dinner alone and being overweight because it was a cross-sectional study. Therefore, the possibility of reverse causality cannot be denied.

## Conclusions

The present study found that eating dinner alone is significantly associated with being overweight among adolescent girls, but there was no statistically significant relationship between eating dinner alone and being overweight among adolescent boys in this community in Japan. Therefore, reducing the frequency of eating dinner alone might contribute to decreasing the risk for being overweight or obese among adolescent girls.

## Abbreviations

BMI: Body mass index
CI: Confidence interval
OR: Odds ratio
SD: Standard deviation
